# Application of multivariate statistical approach to identify trace elements sources in surface waters: a case study of Kowalskie and Stare Miasto reservoirs, Poland

**DOI:** 10.1007/s10661-017-6089-x

**Published:** 2017-07-01

**Authors:** Marcin Siepak, Mariusz Sojka

**Affiliations:** 1 Department of Hydrogeology and Water Protection, Adam Mickiewicz University, 12 Bogumiła Krygowskiego Street, 61-680 Poznań, Poland; 2 Subdepartament of Hydrology and Water Resources, Poznań Life Science University, 94 Piątkowska Street, 61-691 Poznań, Poland

**Keywords:** Trace elements, Pollution, Urban sources, Agricultural catchment, Multivariate statistical techniques, ICP-QQQ

## Abstract

The paper reports the results of measurements of trace elements concentrations in surface water samples collected at the lowland retention reservoirs of Stare Miasto and Kowalskie (Poland). The samples were collected once a month from October 2011 to November 2012. Al, As, Cd, Co, Cr, Cu, Li, Mn, Ni, Pb, Sb, V, and Zn were determined in water samples using the inductively coupled plasma with mass detection (ICP-QQQ). To assess the chemical composition of surface water, multivariate statistical methods of data analysis were used, viz. cluster analysis (CA), principal components analysis (PCA), and discriminant analysis (DA). They made it possible to observe similarities and differences in the chemical composition of water in the points of water samples collection, to uncover hidden factors accounting for the structure of the data, and to assess the impact of natural and anthropogenic sources on the content of trace elements in the water of retention reservoirs. The conducted statistical analyses made it possible to distinguish groups of trace elements allowing for the analysis of time and spatial variation of water in the studied reservoirs.

## Introduction

In recent years, there has been increased interest in trace elements in surface waters due to their toxicity, long-term occurrence in the water ecosystem, accumulation in water plant and animal organisms, as well as the fact that they can be transferred to people’s digestive system and consequently cause health problems (Cook et al. 1990; Deniseger et al. 1990; Sin et al. 2001). Trace elements penetrate to surface waters from natural and anthropogenic sources (Krishna et al. 2009; Férauda et al. 2009; Zhao et al. 2011; Walna and Siepak 2012; Zeng et al. 2015). In non-contaminated water reservoirs, the concentrations of most trace elements are low and originate mainly from the weathering of parent rock and soil (Reza and Singh 2010).

The contamination of surface waters with trace elements tends to occur in areas that are highly urbanized, industrialized, or systematically supplied with agricultural land. This especially refers to the areas where the pH of soil has decreased and the areas which have been undergoing uncontrolled fertilization over many years with fertilizers and pesticides containing high concentrations of trace elements (Dudka and Adriano 1997; Martin 2000; Abollino et al. 2002; Helios-Rybicka et al. 2005; Ettler et al. 2006; Macklin et al. 2006; Nouri et al. 2008; Reza and Singh 2010; Ibragimow et al. 2010, 2013).

Trace elements penetrating to surface water from natural or anthropogenic sources are distributed during their transport between the aqueous phase and bed sediments (Bradley and Cox 1986; Horowitz 1991; Jha et al. 2002; Sobczynski et al. 2004; Gaur et al. 2005). It has been estimated that over 90% of trace elements content in surface water is adsorbed on the suspension and then undergoes sedimentation to bottom sediments (Adamiec and Helios-Rybicka 2002; Helios-Rybicka et al. 2005).

The concentration levels of many elements occurring in surface water usually reach the values in micrograms per liter (ppb) to milligrams per liter (ppm) (Siepak et al. 2004; Walna and Siepak 2012; Yu et al. 2012; Wang and Zang 2014). Consequently, analytical techniques characterized by very low detection limits are required for their determination. Historically, atomic absorption spectrometry (ASA) with different type of atomization was used for the determination of trace elements in water. It is a traditional single-element analytical technique characterized by high time consumption related to performing the determinations and prone to background correction interferences in some sample matrices (Miller-Ihli and Baker 2001). The instrumental techniques available now for environmental analysis have reached a high degree of sensitivity for the most chemical elements. They are increasingly rapid and reliable, meaning that a battery of tests can be carried out in a very short time (Vazquez et al. 2004).

At present, the increasingly common techniques applied in the determination of trace elements in surface waters include inductively coupled plasma optical emission spectrometry (ICP-OES) and inductively coupled plasma mass spectrometry (ICP-MS) (Barałkiewicz et al. 2008; Skorek et al. 2012), characterized by low detectability limits (even at the level of pg/L), high determination precision (below 1% RSD), wide range of calibration curves straightness, and short analysis time compared with other spectroscopic techniques.

Multivariate statistical techniques, such as cluster analysis (CA), principal component analysis (PCA), factor analysis (FA), and discriminant analysis (DA), were applied to evaluate time and spatial variations of water quality data sets for river, lake, and dam reservoirs. Usefulness of multivariate statistical techniques for the evaluation and interpretation of complex data sets, apportionment of pollution sources and factors, and the design of a monitoring network for the effective management of water resources was conducted (Kazi et al. 2009; Li and Zhang 2010; Varol et al. 2012; Wang et al. 2012; Zhao et al. 2012; Cavalcante et al. 2013; Li et al. 2013; Varol 2013). These methods are used to reduce the number of variables. The reduced set of variables may assist in the identification and description of spatial patterns in water quality that result from hydrologic and geochemical processes and from sources of contamination (Olsen et al. 2012).

The main study aims were as follows: (1) to apply chemometric techniques (CA, PCA) in order to describe similarities or possible differences between the contents of trace elements in particular water sampling points, (2) to define anthropogenic or natural sources of trace elements in water, (3) to apply DA in order to distinguish groups of trace elements necessary for analyzing time and space variation of water in reservoirs.

## Material and methods

### Study area

Poland is a country with relatively small water resources. These resources have high spatial and temporal variability. The greatest water deficits are observed most frequently in the central part of Poland, especially in the Wielkopolska region. In order to reduce the water deficit in the Wielkopolska region, 36 reservoirs were built. During the last 50 years, the approach to planning and design of reservoirs has changed. In the initial period, the storage reservoirs were constructed as single-part water bodies. Nowadays, two-stage storage reservoirs are preferred. Such solutions enable the reduction of sediment deposition and better control of the water quality in the main part (Dysarz and Wicher-Dysarz 2013). The Kowalskie and Stare Miasto reservoirs were chosen as the study object on the basis of the above factors.

**Fig. 1:**
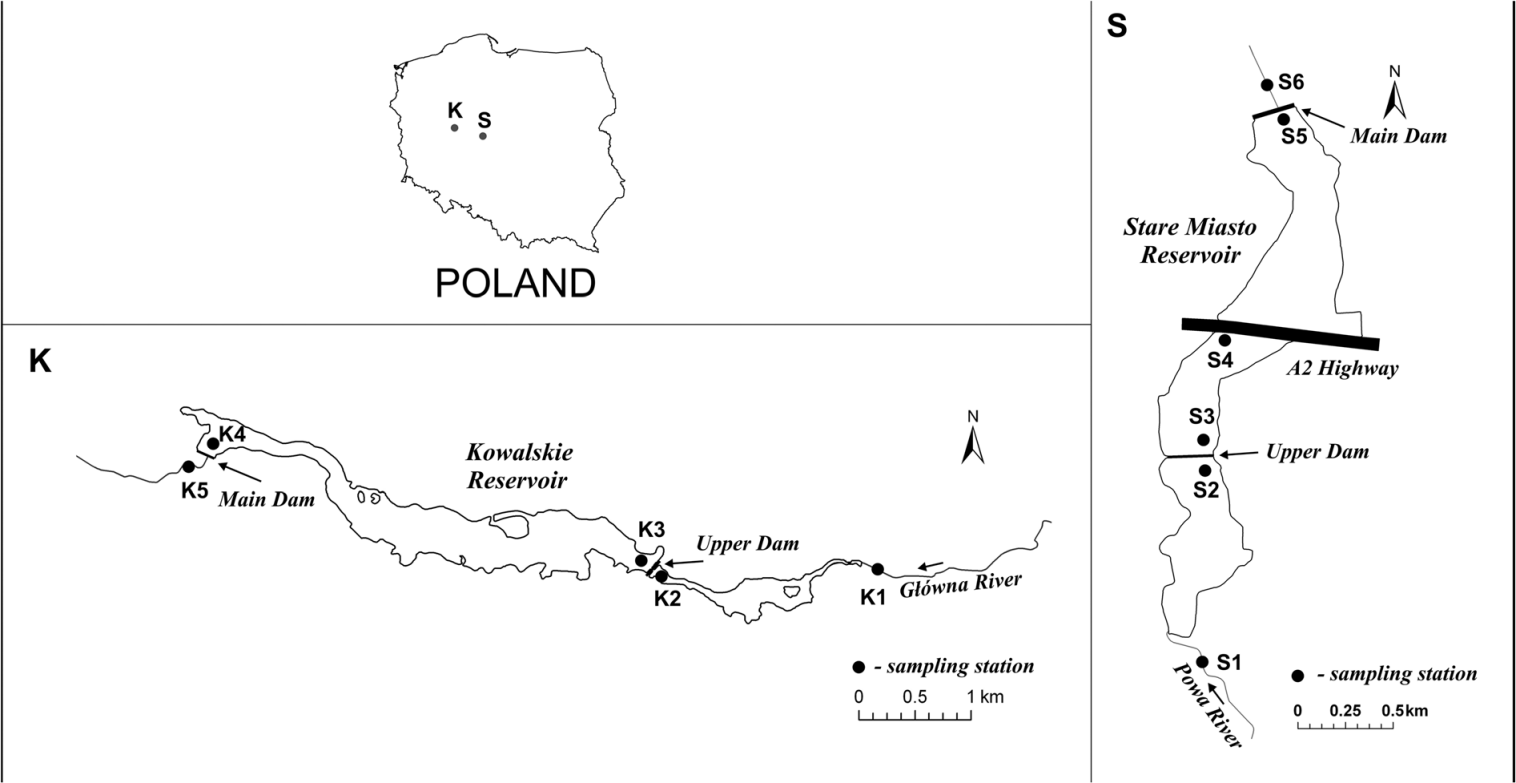
Location of the sampling station in Stare Miasto and Kowalskie reservoirs

Kowalskie and Stare Miasto are artificial retention reservoirs located in the central part of Poland (Fig. 1). The reservoirs are characterized by a specific shape of bowl, as so-called pre-dam reservoirs were separated from them. The pre-dam reservoir, located in the upper part, is smaller than the lower reservoir and functions as a settling tank. The lower part, called the main reservoir, contains the basic functional capacities. Such a solution is one of the methods to prevent the accumulation of debris and degradation of water quality in retention reservoirs. The main parts of Kowalskie and Stare Miasto reservoirs are filled up to the normal accumulation level (NPP), which is maintained until the end of October. In October, the reservoirs are emptied.

Kowalskie reservoir is located on the Główna River, it was commissioned in 1984. The reservoir area is 203 ha (with NPP = 87.00 m ASL), and its total capacity amounts to 6.580 mln m^3^. The area of pre-dam reservoir is 40.4 ha and its capacity amounts to 0.590 mln m^3^. The reservoir depth ranges between 1.5 m and about 6.5 m at the upper dam. The mean annual flow of the Główna River at the Wierzenica profile (*A* = 222 km^2^) located 1 km below the reservoir amounted to 0.73 m^3^·s^−1^ in the years 1977–2009. The mean water retention time in the initial tank is about 9 days. In terms of the usable structure of the catchment, agricultural land is predominant, while arable land takes up about 62.8%, grass land 7.4%, and orchards 1.2%. In the studied catchment, about 20.4% are forests, 4.0% is surface water, and 0.1% swamps. The participation of built-over area is small and amounts to 4.2%.

Stare Miasto reservoir is located on the Powa River, it was commissioned in 2006. The reservoir length is 4.5 km, and the flood area at normal accumulation level (NPP = 93.50 m ASL) amounts to 90.68 ha. The total capacity of the reservoir amounts to 2.159 mln m^3^.

The area of pre-dam reservoir in Stare Miasto reservoir is 27 ha and its capacity amounts to 0.590 mln m^3^. The main reservoir is intersected by the A2 motorway, which additionally divides it into two parts. The depth of the reservoir ranges between 1.2 m and about 5.7 m at the upper dam. The mean flow (SSQ) of the Powa River at the Posoka profile (*A* = 332 km^2^) located below the reservoir reached 1.17 m^3^·s^−1^ in the years 1975–2009. The mean time of water retention in the initial tank is about 3 days. In terms of the usable structure of the catchment, agricultural land is predominant, while arable land takes up about 52%, grass land 14.4%, and orchards 1.6%. In the studied catchment, about 27.6% are forests, 0.6% is surface water, and 0.7% swamps. The participation of built-over area is small and amounts to 3.1%.

### Sample collection

Surface water was sampled for trace elements analysis from October 2011 to November 2012 in 11 gauging cross-sections situated along the reservoirs. The cross-sections were situated in Kowalskie and Stare Miasto reservoirs (Fig. 1). Due to the specific shape of retention reservoirs, water for analyses was sampled above the reservoir, as well as above and below the central and main dam. Due to the fact that Stare Miasto reservoir is intersected by the A2 motorway, one sampling point was additionally localized below.

The samples with volume of 100 mL were collected in polyethylene bottles (HDPE) produced by Nalgene® using a Toń 2 sampler (Mera Błonie, Gdańsk, Poland) and preserved with 0.5 mL of 60% HNO_3_ Ultrapur® and CHCl_3_ (both Merck; Darmstadt, Germany). The water temperature, pH, and electrolytic conductivity were measured directly at the sampling sites using a multi-function Multi 197i device equipped with the electrodes: pH-Electrode SenTix 41 and TetraCon® 325 produced by WTW (Weilheim, Germany). After sampling, the samples were taken to the chemical laboratory in a mobile refrigerator at a temperature of 4 ± 2.5 °C. Adequate precautions were exercised to avoid contamination of water during sampling, transport, and handling.

### Chemical analysis

Concentrations of Al, As, Cd, Co, Cr, Cu, Li, Mn, Ni, Pb, Sb, V, and Zn were determined by ICP-MS. The Agilent 8800 Triple Quad (Agilent Technologies, Japan) was used. The elements were determined according to the International Standard of water quality (ISO 2003). The instrumental equipment and the operating parameters are listed in Table 1. The apparatus was calibrated using a commercially available multi-element model produced by VHG Labs (Manchester, USA). The water samples were also analyzed for their cations (Ca^2+^ and Mg^2+^) and anions (Cl^−^ and SO_4_^2−^) by employing a Metrohm ion chromatograph (IC), model 881 Compact IC Pro (Metrohm, Switzerland).

**Table 1:** Instrumental characteristics and setting for ICP-QQQ

Spectrometer	Agilent 8800 Triple Quad
Nebulizer	MicroMist
Interface	Sampler and skimmer cones in Ni
RF power	1550 W
RF matching	1.80 V
Plasma flow rate (L/min)	15
Carrier gas flow (L/min)	1.05
Nebulizer pump (rps)	0.30
Sample depth (mm)	8.0
Gas flow rate	
	He 4.0 mL/min (purity ≥99.9999%)
	O_2_ 30% (purity ≥99.9999%)
Internal standard	^45^Sc, ^89^Y, ^159^Tb, and ^209^Bi

The reagents used were ultrapure, and the water was de-ionized to a resistivity of 18.2 MΩ·cm in a Direct-Q® 3 Ultrapure Water System apparatus (Millipore, France). Analytical quality control was verified by the analysis of certified reference material for water SRM 1643e (National Institute of Standards & Technology, USA). The obtained results are presented in Table 2.

**Table 2:** Comparison of certified mass concentration and determination value (μg/L)

Element	Certified value	Obtained value
	NIST 1643e	ICP-QQQ
Al	141.8 ± 8.6	141.3 ± 2.3
As	60.45 ± 0.72	60.72 ± 0.28
Cd	6.568 ± 0.073	6.501 ± 0.099
Co	27.06 ± 0.32	27.84 ± 1.19
Cr	20.40 ± 0.24	20.55 ± 0.65
Cu	22.76 ± 0.31	22.54 ± 0.30
Li	17.4 ± 1.7	17.4 ± 0.1
Mn	38.97 ± 0.45	39.03 ± 0.13
Ni	62.41 ± 0.69	62.47 ± 0.65
Pb	19.63 ± 0.21	19.34 ± 0.50
Sb	58.30 ± 0.61	58.49 ± 0.41
V	37.86 ± 0.59	39.01 ± 0.85
Zn	78.5 ± 2.2	78.8 ± 0.31

Average values ± standard deviations

### Data treatment and statistical analysis

The statistical analysis involved the attempt to apply multivariate statistical methods for CA and PCA to determine the sources of trace elements in the water of the studied retention reservoirs, with separated pre-dam reservoir, located in the central part of Poland.

The methods of CA and DA were also applied to assess spatial and time variation of trace elements in the water of the reservoirs. The basis for the assessment was a set of data comprising the determinations of 13 trace elements in the water of two retention reservoirs: Kowalskie and Stare Miasto, along which water was sampled in five and six measurement and control profiles in the period from October 2011 to November 2012.

During the first stage of statistical analysis, the content of trace elements in retention reservoirs was generally characterized. The minimal, maximal, mean, and median values of trace elements concentrations were determined in the water in both reservoirs. Box and whisker plots were drawn up, with the marked values of median, lower 25% and upper 75% quartiles, and extreme values. Analysis of variance (ANOVA) was used to test the significant spatial and temporal differences.

The second stage of statistical analysis involved the analysis of trace elements concentrations at sampling points using multivariate statistical methods: CA, PCA, and DA (Statistica 10 software).

Multivariate statistical methods were conducted on measured data standardized through *z*-scale transformation in order to avoid misclassification due to wide differences in data dimensionality. The autoscaling involved bringing the variances of all determined trace elements to a common value of one.

In order to determine mutual similarities or possible differences in the concentrations of trace elements at particular water sampling points, as well as the relationship between particular elements, CA was conducted. Hierarchical agglomerative CA using the squared Euclidean distance with Ward’s method was applied.

Principal components analysis allowed for the reduction of 13 determined trace elements originally applied to describe the condition of water in retention reservoirs to the new, mutually orthogonal variables, that is principal components, without substantial loss of information included in the data. In order to facilitate interpretation of the results, non-zero factors were rotated by normalized VARIMAX method. The varimax normalized factor rotation was used to maximize the sums of variances of required loadings of the factor matrix and redistribute the variance from earlier factors to later ones to achieve a simpler, theoretically more meaningful factor pattern (Simeonov et al. 2007).

The principal components analysis made it possible to define the origins of trace elements in the reservoirs and to describe their time variation (Singh et al. 2005, 2006; Shrestha and Kazama 2007; Sojka et al. 2008; Zhang et al. 2013; Mostafaei 2014). It was assumed that the principal components significant for the description of data structure were the ones with eigen value higher than 1. Apart from this, it was assumed that when factor loadings between the concentrations of selected trace elements and principal components are 0.75–1.00, 0.50–0.75, and 0.30–0.50, they are adequately powerful, averagely, and weakly correlated (Liu et al. 2003).

DA is used to determine the variables which discriminate between two or more naturally occurring groups. It operates on raw data and the technique constructs a discriminant function for each group. In this study, the following groups have been selected: two and three groups for spatial analysis (sampling group two and three for Kowalskie and Stare Miasto, respectively—groups determined based on CA) and two groups for temporal analysis (water management seasons in reservoir—groups formed based on the water management manual). DA was applied to raw data by using the standard, forward, and backwards stepwise modes to construct discriminant functions to evaluate spatial and temporal variations of trace elements in reservoir and to identify the most significant discriminating variables.

## Results and discussion

The results revealed that in most of the sampling stations, the water was slightly alkaline. The pH of the studied water ranged from 7.62 to 9.30 for the Kowalskie reservoir and from 7.19 to 9.08 for the Stare Miasto reservoir. It was also observed that the water pH was higher in the winter period than in the summer. The above values usually indicate the presence of carbonates of calcium and magnesium in water (Begum et al. 2009; Reza and Singh 2010). The high pH of the water may result in the reduction of trace elements (Aktar et al. 2010). In the case of EC, higher values were found in the water of Kowalskie reservoir (459–1083 μS/cm) than in the water of the Stare Miasto reservoir (255–454 μS/cm). Similarly higher concentrations of basic anions and cations were found in the water of Kowalskie reservoir (50–90 mg Cl^−^/L, 88.9–174.5 mg SO_4_^2−^/L, 68–116 mg Ca^2+^/L, 4.86–31.6 mg Mg^2+^/L) than in the water of Stare Miasto reservoir (25–40 mg Cl^−^/L, 33.3–115.2 mg SO_4_^2−^/L, 40–76 mg Ca^2+^/L, 4.85–19.5 mg Mg^2+^/L).

Concentrations of trace elements in the water of Kowalskie and Stare Miasto reservoirs were compared and presented in Table 3. Average concentrations of trace elements in Kowalskie retention reservoir can be ordered in the following descending order: Mn > Al > Zn > Cu > Li > Ni > As > Pb > Sb > V > Co > Cr > Cd. In Stare Miasto reservoir, the concentration of elements could be presented in a similar way, only the concentrations of Sb, V, and Co were ordered slightly differently. The results of analyses showed that the contents of trace elements in retention reservoirs generally exceeded the geochemical background values for the studied area.

**Table 3:** Statistical summary of selected metal concentrations in water samples in Kowalskie and Stare Miasto reservoirs

Metals	Kowalskie Lake reservoir (μg/L, *n* = 54)					Stare Miasto reservoir (μg/L, *n* = 71)				
	Range		Mean	Median	SD	Range		Mean	Median	SD
Al	2.86	88.9	17.0	13.2	14.8	5.82	471.9	38.6	26.2	65.9
As	0.64	2.81	1.59	1.61	0.55	0.32	6.89	2.07	1.76	1.29
Cd	0.01	0.25	0.06	0.05	0.05	0.01	0.42	0.08	0.05	0.07
Co	0.179	0.841	0.356	0.310	0.139	0.158	1.050	0.369	0.336	0.132
Cr	0.077	0.507	0.180	0.169	0.076	0.072	0.880	0.223	0.191	0.135
Cu	1.34	36.9	7.09	4.14	8.22	1.04	24.9	6.45	5.13	4.99
Li	3.14	5.40	3.83	3.63	0.59	2.47	4.16	3.18	3.16	0.32
Mn	18.7	474.5	136.2	100.7	94.7	84.6	1187.2	267.9	215.4	183.3
Ni	0.95	18.4	3.25	2.55	2.86	1.09	8.56	2.64	2.28	1.36
Pb	0.30	3.34	1.27	1.19	0.68	0.32	4.23	1.55	1.28	0.98
Sb	0.11	14.3	1.15	0.25	2.77	0.07	1.26	0.30	0.22	0.25
V	0.31	0.88	0.61	0.61	0.11	0.33	2.45	0.69	0.60	0.35
Zn	2.61	46.9	14.4	10.9	10.6	3.88	83.9	16.9	12.2	13.9

**Fig. 2:**
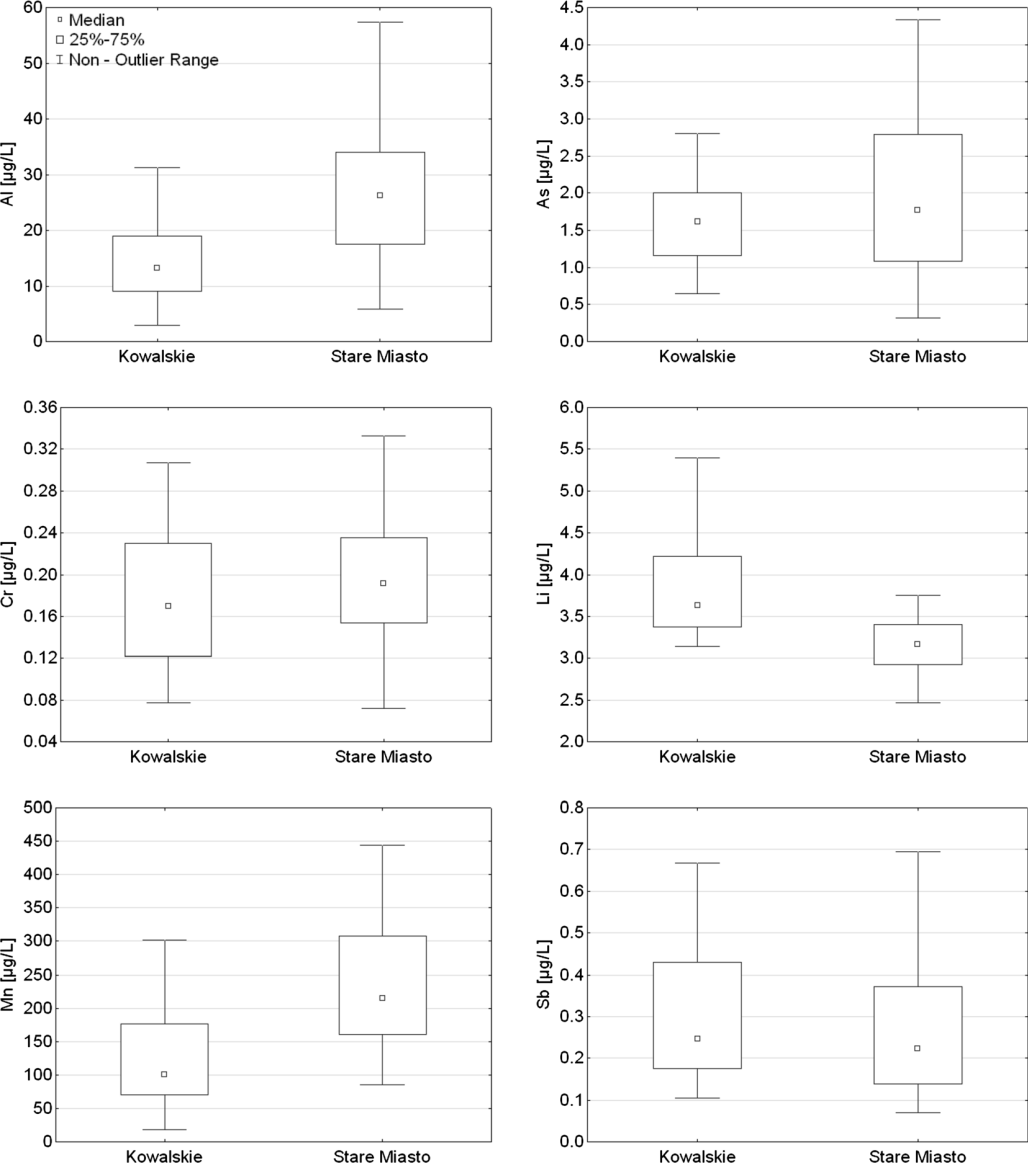
Box and whisker plots for the concentration of Al, As, Cr, Li, Mn, and Sb

Concentrations of selected trace elements in Kowalskie and Stare Miasto reservoirs are presented in box whisker plots (Fig. 2). The analysis of mean values variation significance revealed higher concentrations of Al, As, Cr, and Mn in Stare Miasto reservoir. A different situation was observed for Li and Sb, whose higher concentrations were usually determined in Kowalskie reservoir (Fig. 2). In the case of the remaining trace elements Cd, Co, Cu, Ni, Pb, V, and Zn, their concentrations in both reservoirs were at a similar level. The concentrations of Al, As, Cd, Cr, Mn, Pb, V, and Zn were marked by higher variation in Stare Miasto reservoir, while Cu, Li, Ni, and Sb—in Kowalskie reservoir.

**Fig. 3:**
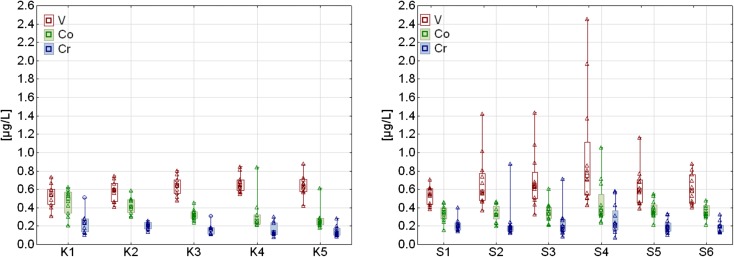
Changes in Co, Cr, and V concentration in Kowalskie and Stare Miasto reservoirs

The analysis of spatial variations of trace element concentrations revealed the decrease in Li, Co, Cr, Al, and Mn and the increase in V contents at the sampling points located along Kowalskie reservoir. A slightly different situation was observed in Stare Miasto reservoir, along which only the decrease in Li content and increase in As content was found. The decrease in trace elements concentration along the retention reservoirs should be linked to the sedimentation of debris floating within the reservoir bowl, which is to the greatest extent responsible for their transportation in water ecosystems. The example changes in Cr, Co, and V contents at the sampling points in Kowalskie and Stare Miasto reservoirs are presented in Fig. 3.

The lack of clear spatial tendency of trace elements along Stare Miasto reservoir was caused by the increase in Al, Cr, Co, Mn, and V concentrations at point S4 located in the central part of the reservoir. The increase in trace elements content at this point corresponds with the inflow of pollutants running off the A2 motorway. At the other sampling points—S5 and S6—located below point S4, the decrease in the content of these metals was observed. The analysis of changes in Al, Co, Mn, and V concentrations in Stare Miasto reservoir in the following months was characterized by similar dynamics, which confirms that the elements originate from the same source of contamination.

A similar time variation was also characteristic for the concentrations of Cd, Cu, Ni, Pb, and Zn, whose occurrence corresponds with agricultural activity and point sources of contamination in the studied catchment. The analysis of changes in trace elements concentrations in subsequent months showed that their highest concentration in Stare Miasto reservoir was observed in June, while the lowest concentration occurred in April and September. The content of particular heavy metals in March, May, and September was similar, while in October and November, their high variation was recorded at particular sampling points.

A different situation was observed in Kowalskie reservoir, where the content of trace elements was at the lowest level on the turn of March and April, while the highest values were observed in May and October. The highest dynamics of changes in heavy metal concentrations in the reservoir was observed in May, October, and November. In March, April, and September, only small changes in trace elements content were most often observed in the collected water samples. The analysis of time changes in trace element concentration in the water of both retention reservoirs showed no clear relationship with meteorological and hydrological conditions and water management activities implemented in the reservoirs.

The lowest content of trace elements in the water of both reservoirs was observed in April at the high flows in that period. In turn, high dynamics of changes in element concentrations in October and November results from the lower ability of suspension sedimentation during the draining of reservoirs and shorter time of water retention in the reservoir at minimal accumulation level.

**Fig. 4:**
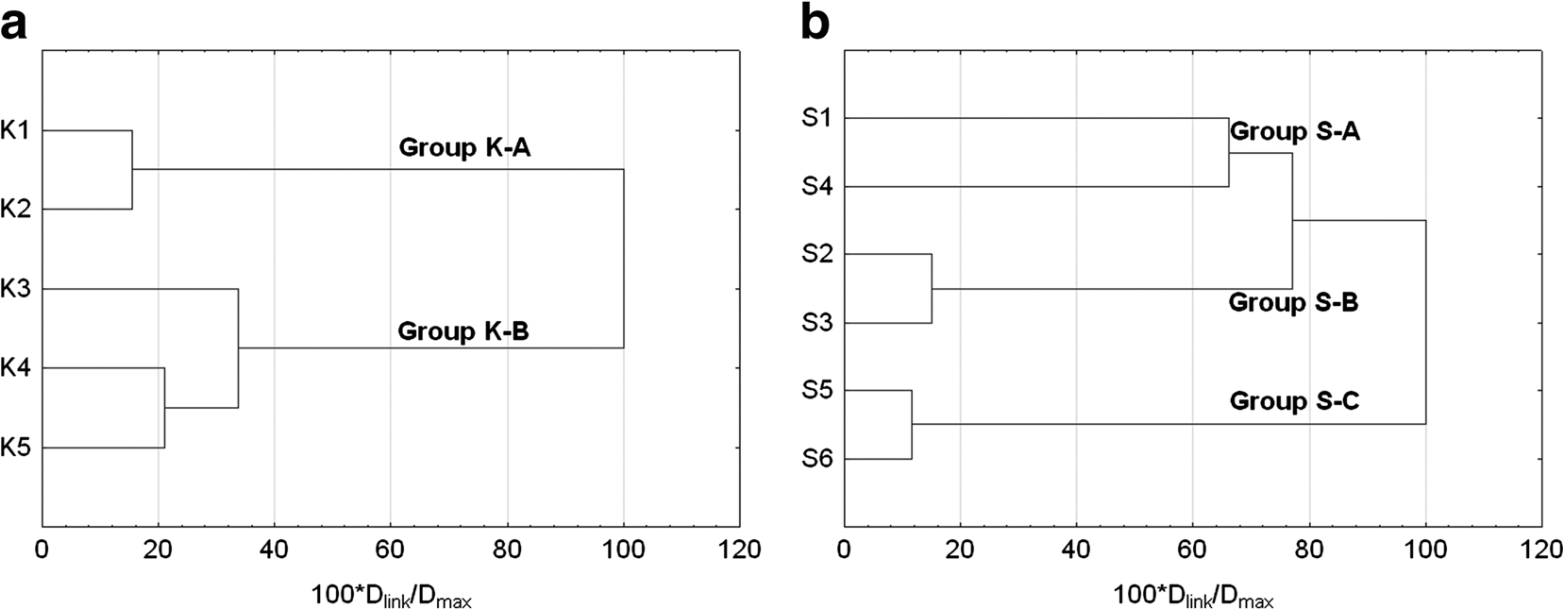
Dendrogram showing clustering of water sampling points in Kowalskie (**a**) and Stare Miasto (**b**) reservoirs

Another stage involved CA conducted in order to determine similarities or possible differences in the content of trace elements at particular sampling points (Fig. 4). The analysis showed that at points K1 and K2 located directly above Kowalskie reservoir and in its pre-dam reservoir the highest value of trace elements was most frequently recorded (group K-A). Lower contents were usually recorded in the main part of retention tank—points K3 and K4, and directly below the front dam—K5 (group K-B) (Fig. 4a).

The content of trace elements was slightly different in Stare Miasto reservoir, where the highest values were recorded at the inflow to the tank at point S1. Another increase in the content of trace elements was recorded at point S4 located near the A2 Highway (group S-A). These points were at the same time characterized by the smallest similarity in terms of the content of particular trace elements, which may be the evidence of the influence of various sources of contamination. The localization of point S1 is affected by contamination originating in different point and non-point sources, while point S4 is under the clear influence of concentrated contamination originating in road traffic. Within the reservoir, two more groups were separated, with a similar level of elements contents.

Near the upper dam separating the pre-dam reservoir from the main reservoir, the concentration of heavy metals was the lowest (group S-B) at points S2 and S3, respectively. In turn, at points S5 and S6 located near the front dam, the content of trace elements in water was average (group S-C) (Fig. 4b).

**Fig. 5:**
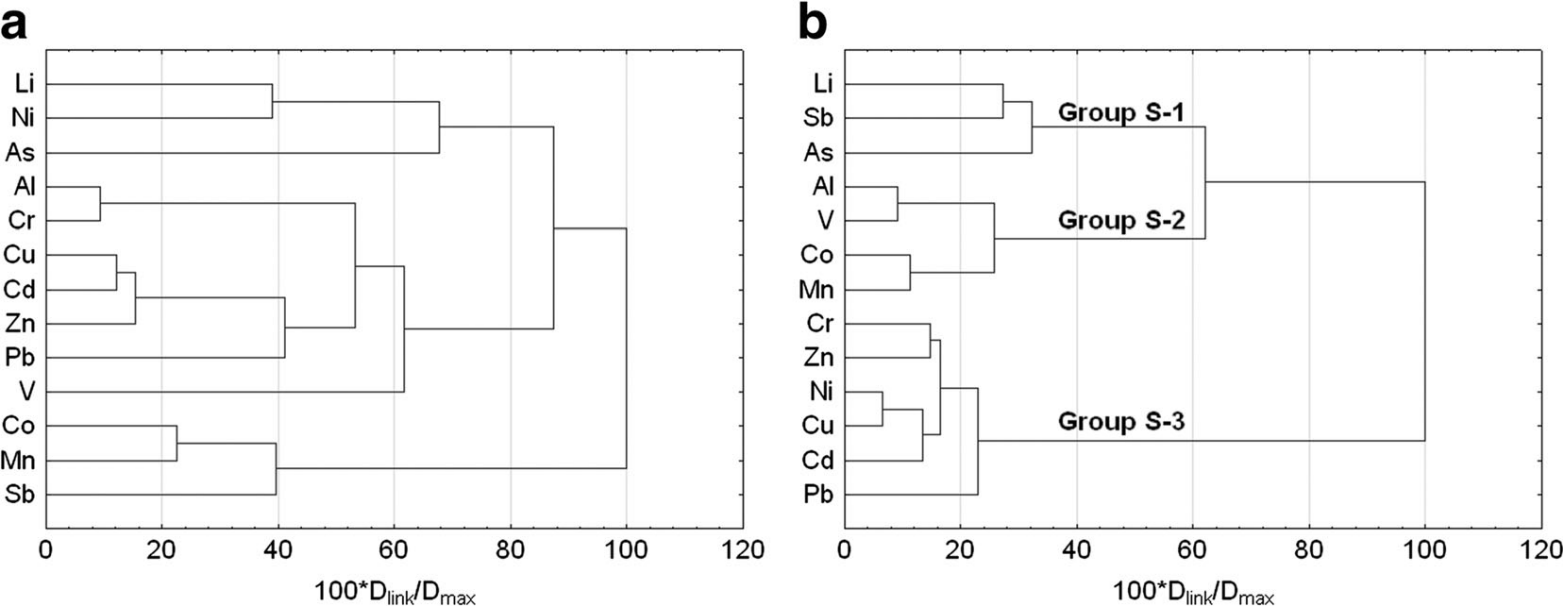
Dendrogram showing clustering of selected metal concentrations in water samples in Kowalskie (**a**) and Stare Miasto (**b**) reservoirs

Cluster analysis did not make it possible to indicate the definite sources of origin of trace elements in Kowalskie reservoir. This results from the simultaneous influence of interpenetrating pollutants from the point and non-point sources of agricultural and urban origins (Fig. 5a).

In the case of Stare Miasto reservoir, three groups of elements were distinguished. Two of them were of anthropogenic origin: Al, V, Co, and Mn from the road traffic (group S-2), Cr, Zn, Ni, Cu, Cd, and Pb, as well as from agricultural activity conducted in the catchment area and point sources of contamination (group S-3). The content of Li, Sb, and As is related to geoenvironmental conditions in the catchment area (geogenic origin) (group S-1) (Fig. 5b).

The analysis of principal components made it possible to reduce the set of 13 trace elements, originally used in the characteristics of water in reservoirs, to four and three principal components in Kowalskie and Stare Miasto reservoirs, respectively. Principal components can also be applied to determine the sources of trace elements in water (Table 4).

**Table 4:** Results of factor analysis for Kowalskie (A) and Stare Miasto (B) reservoirs—factor loadings for three extracted factors, rotation: varimax normalized (loadings >0.75 are bolded and loadings from 0.50 to 0.75 are italics)

Variable	A				B		
	Factor				Factor		
	1	2	3	4	1	2	3
Li	0.09	0.35	**0.78**	−0.08	0.18	−0.04	*0.69*
Al	*0.61*	0.48	0.28	−0.30	0.16	**0.86**	−0.31
V	*0.58*	−0.16	−0.33	−0.26	−0.08	**0.86**	0.11
Co	0.33	**0.84**	0.10	−0.21	0.30	**0.85**	0.02
Cr	0.50	0.54	0.37	−0.33	**0.83**	0.37	−0.16
Mn	−0.14	**0.87**	0.06	0.09	−0.10	**0.73**	0.28
Ni	0.34	−0.18	*0.60*	0.10	**0.91**	0.06	0.09
Cu	**0.87**	0.02	0.29	0.13	**0.88**	−0.13	0.06
Zn	**0.85**	0.26	0.02	0.06	**0.77**	−0.08	0.11
Cd	**0.88**	0.09	0.07	−0.02	**0.81**	0.10	0.03
Sb	0.29	*0.59*	*−0.50*	0.00	0.15	−0.04	*0.63*
Pb	*0.57*	0.19	0.26	0.29	*0.64*	0.08	0.39
As	0.09	−0.10	0.01	**0.91**	−0.24	0.35	*0.66*
Eigenvalues	4.90	2.03	1.46	1.12	4.39	2.87	1.63
% Total variance	37.69	15.61	11.22	8.61	33.80	22.06	12.57
% Cumulative variance	35.69	53.30	64.53	73.14	33.80	55.87	68.43

The extracted factors define 73 and 68% of the structure of primary data and have values higher than 1. In Kowalskie reservoir, the first principal component which defines as many as 37.69% of total variance was additionally strongly correlated with the content of Cu, Zn, and Cd and averagely correlated with Al, V, and Pb contents, which may be the evidence of the natural origin of these components in water. Another three principal components with the accumulated variance of 35.45% were strongly correlated with the concentrations of Co, Mn, Li, and As, respectively.

**Fig. 6:**
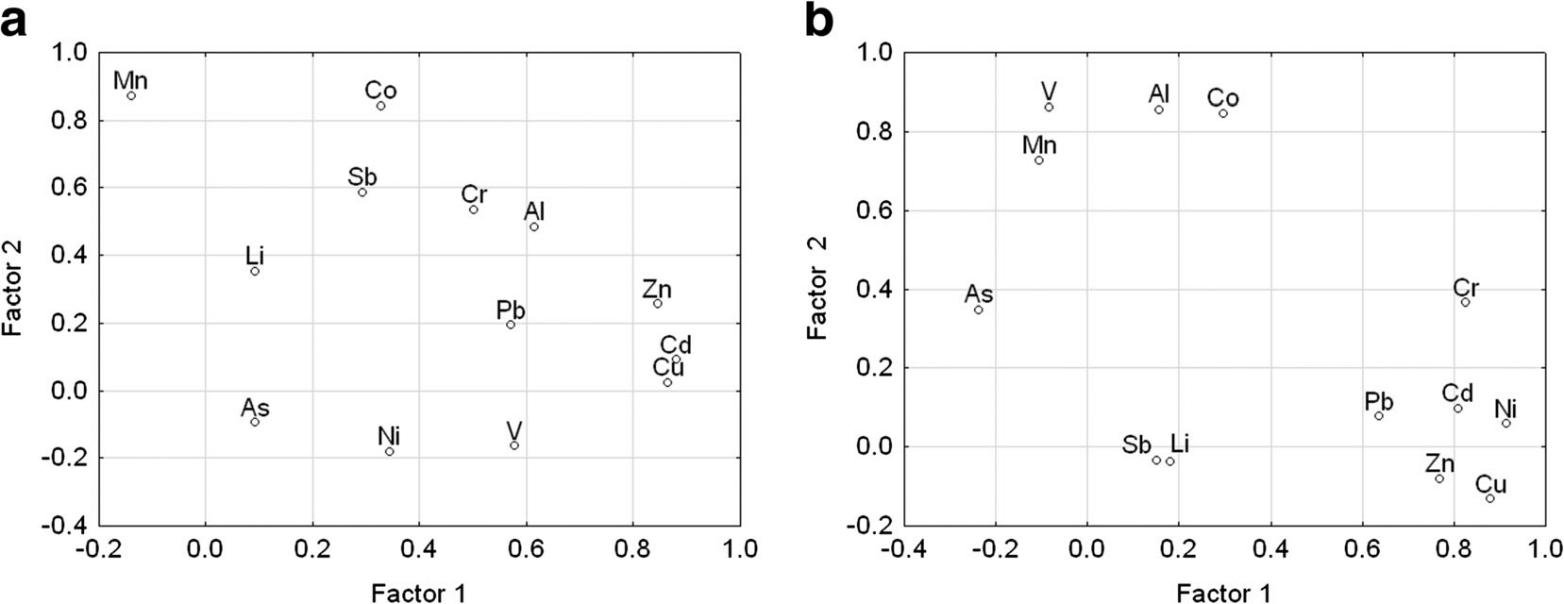
Two-dimensional scatterplot corresponding to the metals in Kowalskie (**a**) and Stare Miasto (**b**) reservoirs determined by F1 and F2

The positive correlation of principal components with the concentrations of selected heavy metals shows that the extracted factors should be linked to geogenic and anthropogenic sources of contamination. In Kowalskie reservoir, the first principal component, whose explained variance is 33.80%, was strongly positively correlated with concentrations of Cr, Ni, Cu, Zn, and Cd, and averagely correlated with Pb concentrations. The remaining two principal components jointly explain 34.63% of data and were strongly correlated with the contents of Al, V, Co, and Mn, as well as Li, components PC2 and PC3, respectively. Principal components analysis confirmed the occurrence of contamination sources of different nature without clear domination of any of them in both catchment areas (Fig. 6).

**Fig. 7:**
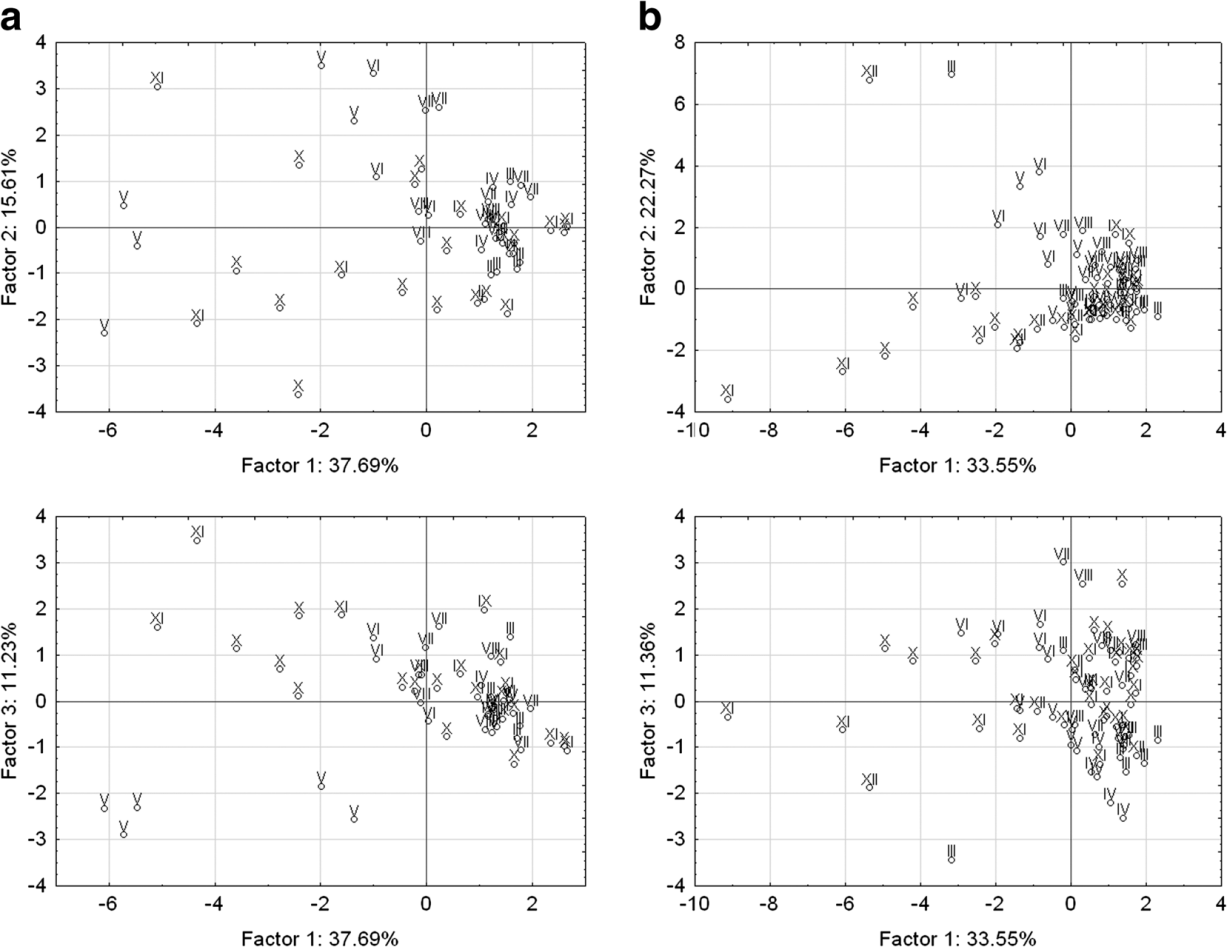
Two-dimensional scatterplot corresponding to the months in Kowalskie (**a**) and Stare Miasto (**b**) reservoirs determined by F1 and F2

Principal components analysis performed by VARIMAX method with rotation did not allow for the definite characteristics of time variation of trace elements content in the water of both reservoirs (Fig. 7). This is due to the fact that, in total, the principal components make it possible to interpret only 53.30 and 48.92% of the internal structure of initial parameters in Kowalskie reservoir, and 55.82 and 44.91% in Stare Miasto reservoir. Principal components analysis in Kowalskie reservoir made it possible to clearly distinguish the months of May, October, and November, in which the content of trace elements was at the lowest level and had the lowest variation.

The final stage involved discriminant analysis based on the raw data comprising determinations of 13 trace elements following the grouping of water sampling points, using cluster analysis, into two groups for Kowalskie reservoir (K-A and K-B) and three groups for Stare Miasto reservoir (S-A, S-B, S-C). The results of classification obtained in DA performed by standard method, forward, and backwards stepwise modes are presented in Table 5. In the case of Kowalskie reservoir, discriminant analysis performed by standard method made it possible to form a discriminant function (Dfs), which used all the 13 trace elements.

**Table 5:** Classification matrix for discriminant analysis of spatial variation of trace elements in Kowalskie and Stare Miasto reservoirs

Monitoring regions	% Correct	Spatial assigned DA		Monitoring regions	% Correct	Spatial assigned DA		
		Kowalskie				Stare Miasto		
		K-A	K-B			S-A	S-B	S-C
Standard DA mode								
K-A	85.71	18	3	S-A	50.00	12	5	7
K-B	100.00	0	33	S-B	62.50	5	15	4
				S-C	75.00	1	5	18
Total	94.44	18	36	Total	62.50	18	25	29
Forward stepwise DA mode								
K-A	80.95	17	4	S-A	45.83	11	6	7
K-B	96.97	1	32	S-B	66.67	4	16	4
				S-C	79.17	2	3	19
Total	90.74	18	36	Total	63.89	17	25	30
Back ward stepwise DA mode								
K-A	76.19	16	5	S-A	-	-	-	-
K-B	96.97	1	32	S-B	-	-	-	-
				S-C	-	-	-	-
Total	88.89	17	37	Total	-	-	-	-

This allowed for the correct detection (CMs) of groups K-A and K-B averagely on the level as high as 94%. Slightly lower results were obtained using the forward and backwards stepwise modes—91 and 88%, in which discriminant functions DFs were formed based on five (Li, V, Sb, As, and Ni) and two (Li and V) trace elements, respectively. The analysis of classification results performed using all methods in relation to particular groups K-A and K-B showed that sampling stations located in group K-B (K3, K4, K5) were marked by larger similarity in terms of trace elements content.

Based on the formed discriminant functions, they were determined correctly in 98%, on average. In the case of Stare Miasto reservoir, discriminant analysis performed by standard method allowed for the classification on the level of 62%. The discriminant function in standard method was formed based on all studied trace elements. In the case of forward stepwise mode, the discriminant function was formed on the basis of five parameters (Li, Al, As, V, and Cd) and allowed for the classification of groups distinguished by CA method on the average level of 64%.

In turn, performing classification in backwards stepwise mode in fixed waterside conditions did not allow for the introduction of any variable to the model. The analysis of results obtained for the classification of particular groups showed the largest similarity within group S-C (points S5 and S6), with the results on the level above 75%. The worst classification results were obtained in the case of group S-A (points S1 and S6), which was characterized by the highest variation in trace elements content.

The results of discriminant analysis show that the contents of Li, V, Sb, As, and Ni, as well as Li, Al, As, V, and Cd in Kowalskie and Stare Miasto reservoirs, respectively, are the most significant parameters which allow for spatial characteristics of water sampling points in the reservoirs.

The analysis of time variation of trace elements in water of both reservoirs was also conducted using discriminant analysis for two seasons according to the manual of water management in the reservoirs. Two groups—K-1 and S-1—were distinguished for the period from October to April, and K-2 and S-2 for the period from May to September in Kowalskie and Stare Miasto, respectively.

In the period from May to October, the so-called normal level of accumulation is maintained in the reservoir. The results of discriminant analysis obtained by standard and forward stepwise method allowed for correct classification of both periods on the level of 96%. Discriminant functions were formed based on 13 and 11 trace elements, respectively, determined in the reservoirs. Definitely worse results—on the level of 78%—were obtained using the backwards stepwise mode, in which the discriminant function was formed based on three elements—Li, V, and Co.

In the case of Stare Miasto reservoir, the results of discriminant analysis conducted by standard, forward, and backwards stepwise methods were on the level from 75 to 84%. Discriminant functions were formed based on three parameters for forward stepwise mode, seven parameters for backwards stepwise mode, while all parameters were applied in standard method. In the case of Kowalskie reservoir, it is difficult to clearly define the trace elements whose contents differ significantly in both seasons, while in Stare Miasto reservoir the most significant parameters were, firstly, the contents of Al, Co, and Pb and, secondly, those of Mn, As, Sb, and Li (Table 6).

**Table 6:** Classification matrix for discriminant analysis of time variation of trace elements in Kowalskie and Stare Miasto reservoirs

Monitoring regions	% Correct	Season assigned DA		Monitoring regions	% Correct	Season assigned DA	
		Kowalskie				Stare Miasto	
		K-1	K-2			S-1	S-2
Standard DA mode							
K-1	95.00	19	1	S-1	80.56	29	7
K-2	97.06	1	33	S-2	83.336	6	30
Total	96.30	20	34	Total	81.946	35	37
Forward stepwise DA mode							
K-1	95.00	19	1	S-1	86.12	31	5
K-2	97.06	1	33	S-2	83.34	6	30
Total	96.30	20	34	Total	84.73	37	35
Back ward stepwise DA mode							
K-1	65.00	13	7	S-1	63.89	23	13
K-2	85.29	5	29	S-2	86.11	5	31
Total	77.78	18	36	Total	75.00	28	44

## Conclusions

The results of the determination and analysis of the parameters of trace elements in the period from October 2011 to November 2012 permit the following conclusions:

1. Concentrations of the determined trace elements in retention reservoirs generally exceeded the values of geochemical background.
2. Along Kowalskie reservoir, the decrease in Li, Co, Cr, Al, and Mn content was observed, as well as the increase in the content of V. In Stare Miasto reservoir, only the decrease in Li and increase in As concentrations were found. This was affected by the inflow of contamination running off the motorway.
3. No clear influence of meteorological and hydrological conditions and water management in the reservoirs on the content of trace elements in the water of both retention reservoirs was found.
4. Cluster analysis allowed for distinguishing two and three groups of sampling points, in Kowalskie and Stare Miasto reservoirs, respectively, which were characterized by similar contents of trace elements.
5. In the case of Kowalskie reservoir, cluster analysis did not allow for identification of sources of trace elements in water, which results from interferences of point and non-point contamination sources being the consequence of agricultural activity and urbanization. For Stare Miasto reservoir, a group of elements of geogenic origin and two groups of anthropogenic origin were distinguished.
6. Principal components analysis allowed for the reduction of a set of 13 trace elements originally used in spatial and time variation analysis of water in the reservoirs to four and three principal components in Kowalskie and Stare Miasto reservoirs, respectively.
7. Principal components analysis confirmed the occurrence in both catchment areas of contaminants of different nature, without clear domination of one of them.
8. Discriminant analysis showed that the most significant trace elements allowing for spatial characteristics of water in reservoirs were Li, V, Sb, As, and Ni, as well as Li, Al, As, V, and Cd in Kowalskie and Stare Miasto reservoirs, respectively.
9. In the case of Kowalskie reservoir, the discriminant analysis did not make it possible to clearly select the trace elements whose contents differed significantly in both seasons as a result of water management in the reservoirs. In Stare Miasto reservoir, mainly the contents of Al, Co, and Pb, and secondly those of Mn, As, Sb, and Li differed in both seasons.
